# Extensive telomere erosion is consistent with localised clonal expansions in Barrett’s metaplasia

**DOI:** 10.1371/journal.pone.0174833

**Published:** 2017-03-31

**Authors:** Boitelo T. Letsolo, Rhiannon E. Jones, Jan Rowson, Julia W. Grimstead, W. Nicol Keith, Gareth J. S. Jenkins, Duncan M. Baird

**Affiliations:** 1 Division of Cancer & Genetics, School of Medicine, Cardiff University, Heath Park, Cardiff, United Kingdom; 2 Institute of Cancer Sciences, College of Medical, Veterinary & Life Sciences, University of Glasgow, Glasgow, United Kingdom; 3 GI Cancer Group, School of Medicine, Swansea University, Swansea, United Kingdom; Tulane University Health Sciences Center, UNITED STATES

## Abstract

Barrett’s oesophagus is a premalignant metaplastic condition that predisposes patients to the development of oesophageal adenocarcinoma. However, only a minor fraction of Barrett’s oesophagus patients progress to adenocarcinoma and it is thus essential to determine bio-molecular markers that can predict the progression of this condition. Telomere dysfunction is considered to drive clonal evolution in several tumour types and telomere length analysis provides clinically relevant prognostic and predictive information. The aim of this work was to use high-resolution telomere analysis to examine telomere dynamics in Barrett’s oesophagus. Telomere length analysis of XpYp, 17p, 11q and 9p, chromosome arms that contain key cancer related genes that are known to be subjected to copy number changes in Barrett’s metaplasia, revealed similar profiles at each chromosome end, indicating that no one specific telomere is likely to suffer preferential telomere erosion. Analysis of patient matched tissues (233 samples from 32 patients) sampled from normal squamous oesophagus, Z-line, and 2 cm intervals within Barrett’s metaplasia, plus oesophago-gastric junction, gastric body and antrum, revealed extensive telomere erosion in Barrett’s metaplasia to within the length ranges at which telomere fusion is detected in other tumour types. Telomere erosion was not uniform, with distinct zones displaying more extensive erosion and more homogenous telomere length profiles. These data are consistent with an extensive proliferative history of cells within Barrett’s metaplasia and are indicative of localised clonal growth. The extent of telomere erosion highlights the potential of telomere dysfunction to drive genome instability and clonal evolution in Barrett’s metaplasia.

## Introduction

Barrett’s oesophagus is an acquired, hyper-proliferative and premalignant lesion that arises as a result of prolonged chronic gastroesophageal reflux disease. It leads to the metaplastic replacement of the squamous lining of the lower oesophagus by columnar intestinal-like epithelium and goblet cells [1, 2] and predisposes to the development of oesophageal adenocarcinoma [3, 4]. Barrett’s oesophagus is characterised by genetic heterogeneity; including large-scale copy number changes across the genome, with key loss of heterozygosity (LOH) events at 9p (involving the CDKN2A locus) and 17p (the TP53 locus) that facilitate progression [5, 6]. The loss of TP53 is also considered permissive for the subsequent development of aneuploidy and tetraploidy [7]. This genetic heterogeneity provides the diversity upon which clonal selection can operate and drive progression to adenocarcinoma [8]. The mechanisms that underpin the genetic heterogeneity observed in Barrett’s oesophagus have not been formally identified.

Telomere dysfunction and resulting fusion events are a key mechanism that can drive large-scale genomic instability and clonal evolution in many tumour types [9, 10]. Human telomeres consist of arrays of TTAGGG repeats, which together with the multi-protein complex “shelterin”, cap the ends of the chromosome termini and distinguish the natural chromosome end from internal double-stranded DNA breaks [11]. Telomerase, a reverse transcriptase, maintains telomeres in the germ-line, in some stem cells and 85% of tumours, but is undetectable in most normal somatic tissues [12, 13]. As a consequence, telomeres in normal cells exhibit a progressive decline in telomere length as a function of cell division. Subsequently, telomere erosion triggers replicative senescence, a TP53 dependent cell cycle arrest, considered to provide a tumour suppressive function [14, 15]. Superimposed on gradual telomere erosion are additional mutational events that create short dysfunctional telomeres, in the absence of significant cell division [16, 17]. If DNA damage checkpoints are defective, short telomeres may trigger genomic instability, whereby the loss of the end-capping function leads to telomere-telomere fusion events [18–21] and through anaphase-bridging-breakage fusion cycles generate large-scale rearrangements such as non-reciprocal translocations [22]. Telomere erosion and dysfunction is observed in numerous tumour types including early-stage lesions [10, 23, 24] and the presence, or absence, of telomeres within the length ranges at which fusion can occur is highly prognostic [25, 26]. The development of Barrett’s oesophagus involves a hyper-proliferative and chronic inflammatory state [27]; the associated cell turnover, as a consequence of exposure to reflux acid and inflammatory mediated ROS induction, may drive telomere erosion and dysfunction. Thus telomere dysfunction and fusion may provide one mechanism to create the genetic diversity, upon which selection operates to drive clonal progression in conditions such as Barrett’s oesophagus. Consistent with this, telomere erosion and chromosomal instability are early events in the progression of Barrett’s oesophagus [28, 29] and is associated with LOH at 17p and 9p [30]. Telomere erosion is specific to the epithelium compared with stromal cells [30] but it is not related to levels of telomerase activity [28, 31]. Whilst telomere erosion has been documented previously in Barrett’s oesophagus, it has not clear if telomeres erode close to, or within, the length ranges at which they can become dysfunctional, undergo fusion and hence drive genomic instability. Here, by using high-resolution approaches to determine telomere length, we have sought to examine the full extent of telomere erosion in Barrett’s oesophagus. In doing so, we provide evidence of extreme telomere erosion and clonal evolution. These data are consistent with the view that telomere dysfunction may contribute to the generation of clonal diversity in Barrett’s oesophagus.

## Results

**Fig 1:**
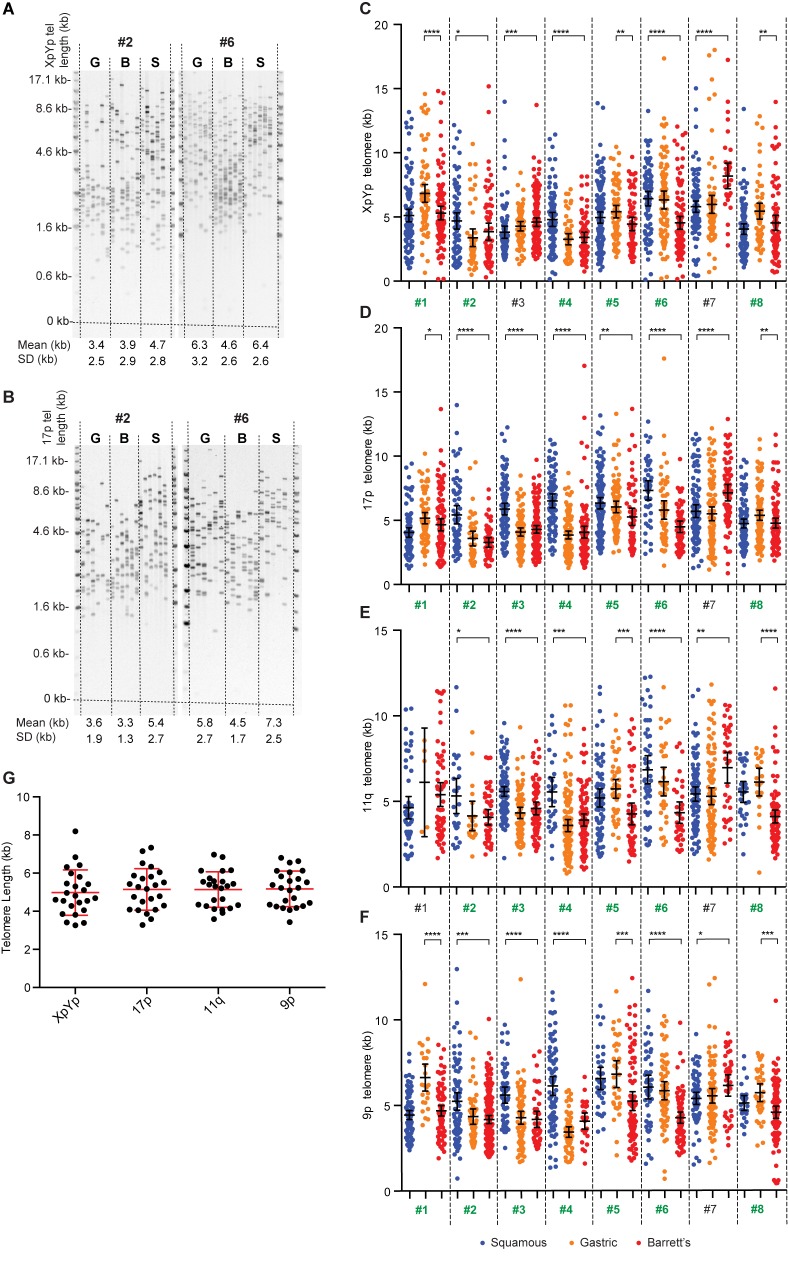
Shorter telomere length profiles are observed in Barrett’s metaplasia but no differences are detected at four different chromosome ends. A, an example of STELA at the XpYp telomere in two patients with matched normal squamous epithelium (S), Barrett’s metaplasia (B) and normal gastric mucosa (G). The mean and standard deviation of telomere length profiles are detailed below. B, example of the same samples analysed with STELA at the 17p telomere. C-F, telomere length profiles obtained from patients #1-#8, as detailed above, depicted as scatter plots obtained by STELA at the telomeres of XpYp (C), 17p (D), 11q (E) and 9p (F). Statistically significant differences between the Barrett’s metaplasia samples with either patient matched squamous or gastric samples are illustrated with asterisks above the plots (two-tailed, Mann-Whitney; * P ≤ 0.05, ** P ≤ 0.01, *** P ≤ 0.001 and **** P ≤ 0.0001), error bars represent 95% confidence intervals. Patients in which the Barrett’s metaplasia sample displayed the shortest, or equal shortest, telomere-length profiles are highlighted in green. G, scatter plot displaying the mean telomere lengths determined for each chromosome end, error bars represent SD.

The loss of loci on 17p (p53), 11q (cyclin D1) and 9p (p16) are known to be related to histological progression in Barrett’s oesophagus [5, 6, 32], we considered that chromosome specific telomere dynamics may drive the loss of loci on specific chromosome arms in Barrett’s oesophagus. To investigate this we examined the telomere length distributions at the chromosome ends of 17p, 11q and 9p, together with XpYp, a chromosome arm that has not been documented to suffer copy-number changes in Barrett’s oesophagus. Analysis was undertaken using the highly sensitive single telomere length analysis (STELA) technique, that is capable of determining the telomere length of specific chromosome ends, and importantly can detect the very short telomeres that are capable of undergoing fusion; these telomeres are not represented in other telomere-length assays [18, 19]. We analysed a small cohort of patients (n = 8) with Barrett’s oesophagus, from which matched samples of gastric mucosae, Barrett’s metaplasia and squamous epithelia had been obtained (Fig 1A and 1B). Despite considerable variation in telomere length among individuals, each individual displayed similar patterns of the telomere length profiles in the tissues and at all four of the chromosome ends analysed (Fig 1A–1F). There was no trend for any one telomere to be significantly shorter than any of the others (p = .51; Fig 1G). It was clear from this analysis that the samples containing Barrett’s metaplasia, exhibited the shortest telomere length profiles in 7 of the 8 individuals analysed (Highlighted in green, Fig 1C–1F). Interestingly however, it was also apparent that in 3 of the patients analysed, the Gastric mucosa and Barrett’s metaplasia displayed indistinguishably short telomere length profiles (Fig 1C–1F). Moreover, the telomere length profiles exhibited substantial heterogeneity, with examples of apparent bimodal distributions observed in some samples, for example patient #2 (Fig 1A); these profiles are consistent with a heterogeneous cellular composition of the samples. Together these data indicate that no one specific telomere is likely to suffer preferential telomere erosion in BE, and whilst Barrett’s metaplasia exhibits telomere erosion, this can also be observed in normal gastric mucosae, with both showing shorter telomere lengths than squamous oesophagus.

**Fig 2:**
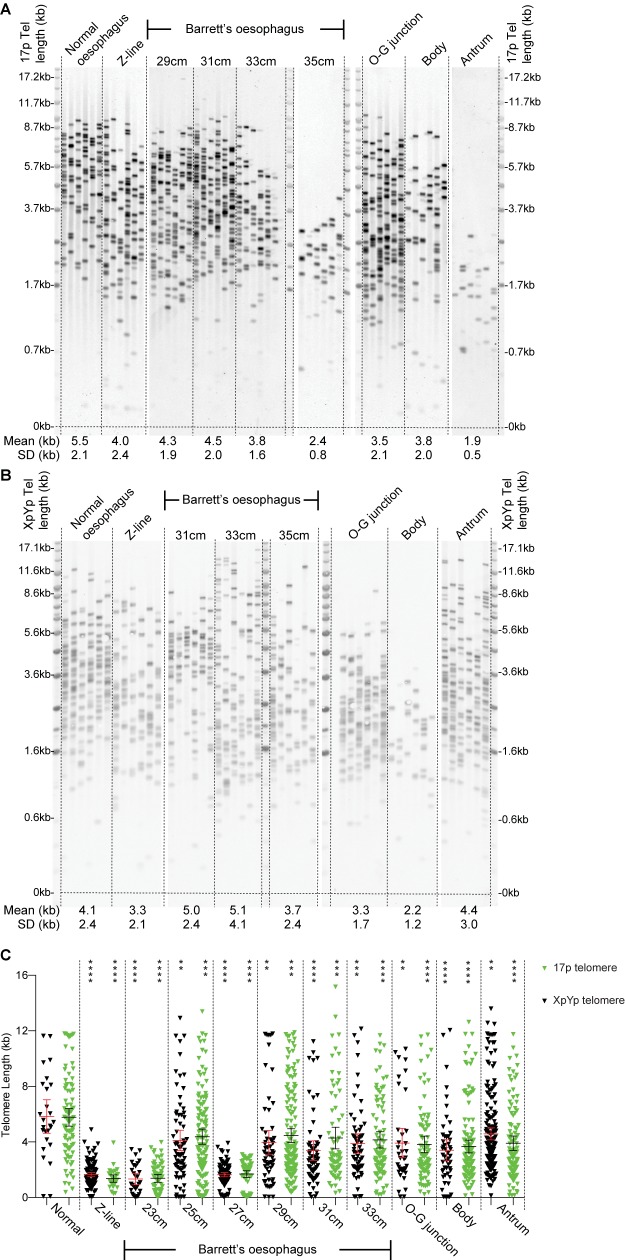
Telomere erosion in Barrett’s metaplasia occurs in zonal patches. A-B, examples of STELA of multiple tissues as indicated above from separate two patients. Mean and standard deviation are detailed below. C, scatterplot depicting STELA data from the XpYp (black) and 17p (green) telomeres from multiple tissues derived from the same patient.

**Fig 3:**
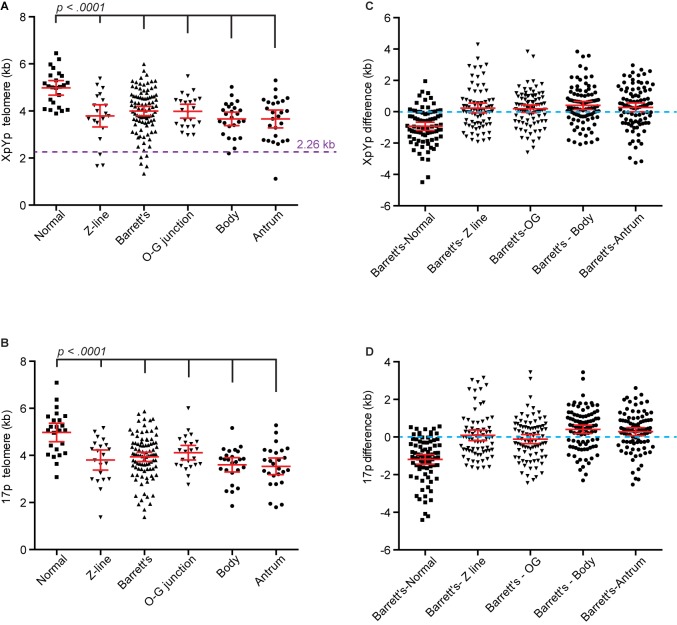
Extensive telomere erosion in Barrett’s metaplasia is similar to that observed in normal gastric tissues. A, mean telomere lengths at the XpYp telomere from the tissues indicated below. The purple dashed line represents the 2.2b kb threshold for prognosis defined in other tumour types [25]. B, mean telomere lengths at the 17p telomere from the tissues indicated below. C-D, scatter plot displaying the difference between Barrett’s metaplasia samples and patient matched normal tissues as indicated at the XpYp telomere (C) and the 17p telomere (D).

In order to examine in more detail the telomere length differentials between different tissues and histological zones within the same tissues, we undertook an analysis of a second cohort of Barrett’s oesophagus patients from which a systematic sampling of tissues at multiple sites throughout both the normal and metaplastic regions of the oesophagus and stomach had been undertaken [31]. Tissues biopsies were taken from normal squamous oesophageal epithelium, the squamo-columnar junction (Z-line), Barrett’s metaplasia at 2 cm intervals, the oesophago-gastric (O-G) junction, the gastric body and the gastric antrum, a total of 209 samples were analysed from 24 patients. Given the within sample homogeneity of telomere length at the four chromosomes analysed in the first cohort, telomere length analysis was undertaken at just the 17p and XpYp telomeres (Fig 2). Comparing the overall means for all the tissue sites it was clear that telomere length in normal squamous oesophageal epithelium was significantly longer than all the other tissues analysed at both chromosome ends (p > .0001; Fig 3A and 3B). Interestingly this included the gastric tissues samples (O-G junction, Body and Antrum) that displayed no significant difference in telomere length compared to those observed in Barrett’s metaplasia (p = .11 and p = .064 for XpYp and 17p; Fig 3A and 3B).

The extent of telomere erosion detected in Barrett’s metaplasia samples was considerable with 21% and 16% of samples exhibiting telomere erosion of over 2 kb compared the matched normal samples at the 17p and XpYp telomeres respectively (Fig 3C and 3D). Moreover, 5% of Barrett’s metaplasia samples, representing 4 of the 24 patients analysed (Fig 3A), exhibited mean telomere lengths that were less than the 2.26 kb threshold that defines a poor prognosis in both Chronic Lymphocytic Leukaemia and Breast Cancer [25, 26]. Strikingly, the telomere erosion observed in Barrett’s metaplasia was not uniform; instead, more extensive erosion was observed in patches at varying distances along the oesophagus (Fig 2), the telomere length within these regions was also more homogenous, consistent with the telomere length profiles observed in single cell clonal populations of primary fibroblast cell cultures [33], or in purified tumour samples as observed in chronic lymphocytic leukaemia [23, 24]. Our observations are consistent with a more extensive proliferative history, with localised clonal expansion within zonal regions of Barrett’s metaplasia, which results in cells containing telomeres within the length ranges at which telomere dysfunction and fusion has been detected.

## Discussion

Our data show that, compared to normal patient-matched squamous epithelium, tissues exhibiting Barrett’s metaplasia display significantly shorter telomeres at all the chromosomes tested. The extent of the difference was considerable with differences in mean telomere length of up to 4.5 kb. If one considers the telomere length profiles of the normal squamous epithelium to be representative of the progenitors from which the Barrett’s metaplasia was formed, then the large telomere length differentials observed are consistent with an extensive replicative history. The telomere dynamics and rates of erosion will be modulated by the replicative kinetics and telomerase activities in these tissues, as well as genotoxic insults that may drive stochastic telomeric shortening. It will thus be difficult to use the telomere length differential between normal and Barrett’s metaplasia to precisely estimate the amount of cell division. However in the absence of telomerase activity, assuming a telomere erosion rate of 85 bp/population doubling as observed in normal telomerase negative human fibroblast populations [33], differences of over 2kb between normal squamous epithelium and Barrett’s metaplasia would represent over 23 cell divisions. Clearly in the presence of telomerase that has been previously documented in Barrett’s metaplasia [31], the rates of telomere erosion will be less and thus the telomere length differentials will represent an even greater number of cell divisions. Moreover, the extent of telomere erosion was not consistent throughout the Barrett’s metaplasia segments, instead we observed distinct differences in both telomere length and the heterogeneity of the distributions within the different zones analysed. Telomere length heterogeneity reflects the clonal composition of the tissue analysed, with more homogeneous distributions indicative of clonal cell populations [33, 34]. Our data indicate that distinct clonal expansions can be observed at different positions throughout the Barrett’s Oesophagus segments and that this varies between patients. Taken together the observations of extensive telomere erosion and clonal expansion are consistent with an extensive replicative history of cells during the development of Barrett’s metaplasia [35].

Our data also show no significant difference in telomere length between the Barrett’s metaplasia and the normal gastric tissues obtained from the OG-junction, the body and antrum. The telomerase activity of these samples has been documented previously [31] and telomerase activity was absent in the gastric tissues; body and antrum. Whereas, all the oesophageal mucosa samples were telomerase-positive and this increased moving distally within the Barrett’s mucosa [31]. Thus the short telomere length distributions observed in gastric tissues, is consistent with on-going cell turnover in absence of sufficient telomerase activity. The presence of short telomeres, within the length ranges that are be capable of undergoing fusion, may account for the high levels of aneuploidy that have previously been documented in normal gastric mucosa [36, 37]. However, caution should be applied to this interpretation, as whilst the ‘normal’ tissues samples were histologically confirmed, they were derived from patients with Barrett’s metaplasia and thus may not be entirely representative of these tissues within the normal population.

Our data demonstrate that telomere erosion does not occur preferentially at specific chromosome ends, with 9p, 11q, 17p and XpYp all displaying similar telomere length profiles. Thus if telomere dysfunction is driving copy number changes at specific chromosome arms then this is not related to the absolute length of the associated telomere. Instead this is more consistent with a situation in which telomere erosion across all chromosome ends leads to the accumulation of short dysfunctional telomeres that are capable of fusion and driving genome-wide copy number changes and selection for specific chromosomal rearrangements [38]. Whilst patient matched genomic data from Barrett’s metaplasia and oesophageal adenocarcinoma indicated a comparatively low level of copy number changes in Barrett’s metaplasia, there was a clear increase in copy number changes within adenocarcinomas [39]; these types of genomic mutation may be consistent with telomere dysfunction during the transition from Barrett’s metaplasia to oesophageal carcinoma. We have previously defined the telomere length ranges in which telomere fusion can be detected in the B-cell clones of patients with Chronic Lymphocytic Leukaemia (CLL). Stratification of both CLL and breast cancer patients based on these thresholds provides definitive independent prognostic information for overall survival [25, 26]. We detected telomere lengths in Barrett’s metaplasia samples within the same length ranges defined in CLL. Mutations within key proteins required for a functional cell cycle checkpoint including p16, p53 and over-expression of cyclin D1 [40–44] are commonly detected in Barrett’s Metaplasia. It is thus tempting to speculate if, in the context of compromised cell cycle checkpoint control, whether telomere-driven genome instability may play a role in driving the progression of patients with Barrett’s Oesophagus.

## Materials and methods

### Sample collections and preparation

Tissue biopsies were obtained from 8 patients undergoing periodic endoscopy at Morriston hospital in Swansea; the tissue samples were composed of gastric mucosae, Barrett’s metaplasia and squamous epithelia. Ethical approval for the collection of samples of Barrett’s oesophagus from endoscopic procedures at Morriston Hospital Swansea, was obtained from the Dyfed Powys local research ethics (LREC) committee prior to commencement of the study. Informed consent was obtained from all patients donating fresh tissue.

A second set of tissue biopsies were obtained from 24 patients undergoing periodic endoscopy for Barrett’s oesophagus at Glasgow Royal Infirmary under conditions of anonymity [31], the study was approved by the Glasgow Royal Infirmary Research Ethics Committee and patients gave written informed agreement to participate. The tissue samples were composed of biopsies taken every 2cm from the normal squamous epithelium of the oesophagus, oesophageal squamo-columnar junction (“Z line”); through Barrett’s metaplasia, gastric cardia or the oesophago-gastric junction, then the gastric body and antrum. All tissue samples were snap frozen in liquid nitrogen and banked at -80°C. The frozen tissues were disrupted with a tissueruptor homogeniser (Qiagen) in lysis buffer after which the genomic DNA was extracted by standard proteinase K, RNase A and phenol/chloroform protocols [45]. High molecular weight DNA was solubilised by digestion in NotI or EcoR1 buffer prior to quantification. The DNA concentration was then estimated in triplicate using Hoechst 33258 fluorometry.

### Single telomere length analysis (STELA)

STELA at the telomeres of XpYp, 17p and 11q was undertaken as described previously [33, 34]. In addition, STELA was also carried out at the telomere of 9p, using the oligonucleotide primer 9p2 (5'-CAC ATT CCT CAT GTG CTT ACG-3'). Multiple PCR reactions (typically 6) were carried out for each DNA sample as follows: 20sec at 94°C, 30sec at 65°C (XpYpE2 [33]), 59°C (17pseq1rev, [34]), 61°C (9p2), and 66°C (11q13B) [34] and 8 minutes at 68°C for 22 (XpYpE2& 17pseq1rev) or 24 (9p2 & 11q13B) cycles. Amplified products were resolved by 0.5% agarose Tris-acetate–EDTA gel electrophoresis and detected by Southern hybridisation with ^33^P-labelled TTAGGG repeat containing probes. This process typically results in 6–10 detectable telomeric molecules in each STELA reaction, however both the concentration and quality of the DNA preparation will affect the dilution and single-molecule amplification efficiency resulting in variable number of amplifiable molecules between samples.

## References

[1] Fass R, Hell RW, Garewal HS, Martinez P, Pulliam G, Wendel C, et al Correlation of oesophageal acid exposure with Barrett's oesophagus length. Gut. 2001;48(3):310–3. 10.1136/gut.48.3.310 11171818 PMC1760147

[2] Haggitt RC. Barrett's esophagus, dysplasia, and adenocarcinoma. Hum Pathol. 1994;25(10):982–93. 7927321 10.1016/0046-8177(94)90057-4

[3] Flejou JF, Svrcek M. Barrett's oesophagus—a pathologist's view. Histopathology. 2007;50(1):3–14. 10.1111/j.1365-2559.2006.02569.x 17204017

[4] Drewitz D, Sampliner R, Garewal H. The incidence of adenocarcinoma in Barrett's esophagus: a prospective study of 170 patients followed 4.8 years. 1997;92:212–5. 9040193

[5] Reid BJ, Prevo LJ, Galipeau PC, Sanchez CA, Longton G, Levine DS, et al Predictors of progression in Barrett's esophagus II: baseline 17p (p53) loss of heterozygosity identifies a patient subset at increased risk for neoplastic progression. Am J Gastroenterol. 2001;96(10):2839–48. 10.1111/j.1572-0241.2001.04236.x 11693316 PMC1808263

[6] Wong DJ, Paulson TG, Prevo LJ, Galipeau PC, Longton G, Blount PL, et al p16(INK4a) lesions are common, early abnormalities that undergo clonal expansion in Barrett's metaplastic epithelium. Cancer Res. 2001;61(22):8284–9. Epub 2001/11/24. 11719461

[7] Rabinovitch PS, Longton G, Blount PL, Levine DS, Reid BJ. Predictors of progression in Barrett's esophagus III: baseline flow cytometric variables. Am J Gastroenterol. 2001;96(11):3071–83. PubMed Central PMCID: PMCPMC1559994. 10.1111/j.1572-0241.2001.05261.x 11721752 PMC1559994

[8] Maley CC, Galipeau PC, Finley JC, Wongsurawat VJ, Li X, Sanchez CA, et al Genetic clonal diversity predicts progression to esophageal adenocarcinoma. Nat Genet. 2006;38(4):468–73. Epub 2006/03/28. 10.1038/ng1768 16565718

[9] Jones CH, Pepper C, Baird DM. Telomere dysfunction and its role in haematological cancer. Br J Haematol. 2012;156(5):573–87. Epub 2012/01/12. 10.1111/j.1365-2141.2011.09022.x 22233151

[10] Roger L, Jones RE, Heppel NH, Williams GT, Sampson JR, Baird DM. Extensive telomere erosion in the initiation of colorectal adenomas and its association with chromosomal instability. J Natl Cancer Inst. 2013;105(16):1202–11. Epub 2013/08/07. 10.1093/jnci/djt191 23918447

[11] de Lange T. Shelterin: the protein complex that shapes and safeguards human telomeres. Genes Dev. 2005;19(18):2100–10. 10.1101/gad.1346005 16166375

[12] Kim NW, Piatyszek MA, Prowse KR, Harley CB, West MD, Ho PL, et al Specific association of human telomerase activity with immortal cells and cancer. Science. 1994;266(5193):2011–5. 7605428 10.1126/science.7605428

[13] Kolquist KA, Ellisen LW, Counter CM, Meyerson M, Tan LK, Weinberg RA, et al Expression of TERT in early premalignant lesions and a subset of cells in normal tissues. Nat Genet. 1998;19(2):182–6. 10.1038/554 9620778

[14] Wright WE, Shay JW. Cellular senescence as a tumor-protection mechanism: the essential role of counting. Curr Opin Genet Dev. 2001;11(1):98–103. 11163158 10.1016/s0959-437x(00)00163-5

[15] Harley CB, Futcher AB, Greider CW. Telomeres shorten during ageing of human fibroblasts. Nature. 1990;345(6274):458–60. 10.1038/345458a0 2342578

[16] Baird DM. Mechanisms of telomeric instability. Cytogenet Genome Res. 2008;122:308–14. 10.1159/000167817 19188700

[17] Murnane JP, Sabatier L, Marder BA, Morgan WF. Telomere dynamics in an immortal human cell line. Embo J. 1994;13(20):4953–62. 7957062 10.1002/j.1460-2075.1994.tb06822.x PMC395436

[18] Capper R, Britt-Compton B, Tankimanova M, Rowson J, Letsolo B, Man S, et al The nature of telomere fusion and a definition of the critical telomere length in human cells. Genes Dev. 2007;21(19):2495–508. 10.1101/gad.439107 17908935 PMC1993879

[19] Letsolo BT, Rowson J, Baird DM. Fusion of short telomeres in human cells is characterised by extensive deletion and microhomology and can result in complex rearrangements. Nucleic Acids Res. 2010;38(6):1841–52. 10.1093/nar/gkp1183 20026586 PMC2847243

[20] Counter CM, Avilion AA, LeFeuvre CE, Stewart NG, Greider CW, Harley CB, et al Telomere shortening associated with chromosome instability is arrested in immortal cells which express telomerase activity. Embo J. 1992;11(5):1921–9. 1582420 10.1002/j.1460-2075.1992.tb05245.x PMC556651

[21] Jones RE, Oh S, Grimstead JW, Zimbric J, Roger L, Heppel NH, et al Escape from Telomere-Driven Crisis Is DNA Ligase III Dependent. Cell reports. 2014;8(4):1063–76. 10.1016/j.celrep.2014.07.007 25127141

[22] Artandi SE, Chang S, Lee SL, Alson S, Gottlieb GJ, Chin L, et al Telomere dysfunction promotes non-reciprocal translocations and epithelial cancers in mice. Nature. 2000;406(6796):641–5. 10.1038/35020592 10949306

[23] Lin TT, Letsolo BT, Jones RE, Rowson J, Pratt G, Hewamana S, et al Telomere dysfunction and fusion during the progression of chronic lymphocytic leukaemia: evidence for a telomere crisis. Blood. 2010;116(11):1899–907. Epub 2010/06/12. 10.1182/blood-2010-02-272104 20538793

[24] Britt-Compton B, Lin TT, Ahmed G, Weston V, Jones RE, Fegan C, et al Extreme telomere erosion in ATM-mutated and 11q-deleted CLL patients is independent of disease stage. Leukemia. 2012;26(4):826–30. Epub 2011/10/12. 10.1038/leu.2011.281 21986843

[25] Lin TT, Norris K, Heppel NH, Pratt G, Allan JM, Allsup DJ, et al Telomere dysfunction accurately predicts clinical outcome in chronic lymphocytic leukaemia, even in patients with early stage disease. Br J Haematol. 2014;167(2):214–23. 10.1111/bjh.13023 24990087

[26] Simpson K, Jones RE, Grimstead JW, Hills R, Pepper C, Baird DM. Telomere fusion threshold identifies a poor prognostic subset of breast cancer patients. Molecular oncology. 2015;9(6):1186–93. PubMed Central PMCID: PMCPMC4449122. 10.1016/j.molonc.2015.02.003 25752197 PMC4449122

[27] Spechler S. Barrett's esophagus. 2002;346:836–42. 10.1056/NEJMcp012118 11893796

[28] Souza RF, Lunsford T, Ramirez RD, Zhang X, Lee EL, Shen Y, et al GERD is associated with shortened telomeres in the squamous epithelium of the distal esophagus. American journal of physiology Gastrointestinal and liver physiology. 2007;293(1):G19–24. Epub 2007/03/31. 10.1152/ajpgi.00055.2007 17395902

[29] Meeker AK, Hicks JL, Iacobuzio-Donahue CA, Montgomery EA, Westra WH, Chan TY, et al Telomere length abnormalities occur early in the initiation of epithelial carcinogenesis. Clin Cancer Res. 2004;10(10):3317–26. 10.1158/1078-0432.CCR-0984-03 15161685

[30] Finley JC, Reid BJ, Odze RD, Sanchez CA, Galipeau P, Li X, et al Chromosomal instability in Barrett's esophagus is related to telomere shortening. Cancer Epidemiol Biomarkers Prev. 2006;15(8):1451–7. Epub 2006/08/10. 10.1158/1055-9965.EPI-05-0837 16896031

[31] Going JJ, Fletcher-Monaghan AJ, Neilson L, Wisman BA, van der Zee A, Stuart RC, et al Zoning of mucosal phenotype, dysplasia, and telomerase activity measured by telomerase repeat assay protocol in Barrett's esophagus. Neoplasia. 2004;6(1):85–92. 15068673 PMC1508632

[32] Galipeau PC, Li X, Blount PL, Maley CC, Sanchez CA, Odze RD, et al NSAIDs modulate CDKN2A, TP53, and DNA content risk for progression to esophageal adenocarcinoma. PLoS Med. 2007;4(2):e67 Epub 2007/03/01 PubMed Central PMCID: PMC1808095. 10.1371/journal.pmed.0040067 17326708 PMC1808095

[33] Baird DM, Rowson J, Wynford-Thomas D, Kipling D. Extensive allelic variation and ultrashort telomeres in senescent human cells. Nat Genet. 2003;33(2):203–7. 10.1038/ng1084 12539050

[34] Britt-Compton B, Rowson J, Locke M, Mackenzie I, Kipling D, Baird DM. Structural stability and chromosome-specific telomere length is governed by cis-acting determinants in humans. Hum Mol Genet. 2006;15(5):725–33. 10.1093/hmg/ddi486 16421168

[35] Barrett MT, Sanchez CA, Prevo LJ, Wong DJ, Galipeau PC, Paulson TG, et al Evolution of neoplastic cell lineages in Barrett oesophagus. Nat Genet. 1999;22(1):106–9. PubMed Central PMCID: PMCPMC1559997. 10.1038/8816 10319873 PMC1559997

[36] Williams L, Jenkins GJ, Doak SH, Fowler P, Parry EM, Brown TH, et al Fluorescence in situ hybridisation analysis of chromosomal aberrations in gastric tissue: the potential involvement of Helicobacter pylori. Br J Cancer. 2005;92(9):1759–66. PubMed Central PMCID: PMCPMC2362026. 10.1038/sj.bjc.6602533 15827559 PMC2362026

[37] Williams L, Somasekar A, Davies DJ, Cronin J, Doak SH, Alcolado R, et al Aneuploidy involving chromosome 1 may be an early predictive marker of intestinal type gastric cancer. Mutat Res. 2009;669(1–2):104–11. 10.1016/j.mrfmmm.2009.05.009 19481101

[38] Liddiard K, Ruis B, Takasugi T, Harvey A, Ashelford K, Hendrickson E, et al Sister chromatid, but not NHEJ-mediated inter-chromosomal telomere fusions, occur independently of DNA ligases 3 and 4. Genome Res. 2016. 10.1101/gr.200840.115 PMC4864465 26941250

[39] Ross-Innes CS, Becq J, Warren A, Cheetham RK, Northen H, O'Donovan M, et al Whole-genome sequencing provides new insights into the clonal architecture of Barrett's esophagus and esophageal adenocarcinoma. Nat Genet. 2015;47(9):1038–46. PubMed Central PMCID: PMCPMC4556068. 10.1038/ng.3357 26192915 PMC4556068

[40] Arber N, Lightdale C, Rotterdam H, Han KH, Sgambato A, Yap E, et al Increased expression of the cyclin D1 gene in Barrett's esophagus. Cancer Epidemiol Biomarkers Prev. 1996;5(6):457–9. Epub 1996/06/01. 8781742

[41] Bani-Hani K. Prospective study of cyclin D1 overexpression in Barrett's esophagus: association with increased risk of adenocarcinoma. 2000;92:1316–21. 10944553 10.1093/jnci/92.16.1316

[42] Jenkins GJ, Doak SH, Parry JM, D'Souza FR, Griffiths AP, Baxter JN. Genetic pathways involved in the progression of Barrett's metaplasia to adenocarcinoma. Br J Surg. 2002;89(7):824–37. 10.1046/j.1365-2168.2002.02107.x 12081731

[43] Hardie LJ, Darnton SJ, Wallis YL, Chauhan A, Hainaut P, Wild CP, et al p16 expression in Barrett's esophagus and esophageal adenocarcinoma: association with genetic and epigenetic alterations. Cancer Lett. 2005;217(2):221–30. Epub 2004/12/25. 10.1016/j.canlet.2004.06.025 15617840

[44] Galipeau PC, Cowan DS, Sanchez CA, Barrett MT, Emond MJ, Levine DS, et al 17p (p53) allelic losses, 4N (G2/tetraploid) populations, and progression to aneuploidy in Barrett's esophagus. Proc Natl Acad Sci U S A. 1996;93(14):7081–4. 8692948 10.1073/pnas.93.14.7081 PMC38939

[45] Sambrook J, Fritsch EF, Maniatis T. Molecular Cloning: a Laboratory Manual. edn n, editor. New York: Cold Spring Harbor Laboratory Press; 1989.

